# Spatial distribution and factors associated with health insurance subscription among women in Ghana

**DOI:** 10.1093/inthealth/ihad092

**Published:** 2023-10-14

**Authors:** Richard Gyan Aboagye, Ebenezer N K Boateng, Yaw Marfo Okyere, Joshua Okyere, Kwamena Sekyi Dickson, Abdul-Aziz Seidu, Bright Opoku Ahinkorah

**Affiliations:** Department of Family and Community Health, Fred N. Binka School of Public Health, University of Health and Allied Sciences, Hohoe, Ghana; Department of Geography and Regional Planning, University of Cape Coast, Cape Coast, Ghana; School of Nursing and Midwifery, University of Cape Coast, Cape Coast, Ghana; Department of Population and Health, University of Cape Coast, Cape Coast, Ghana; Department of Nursing, College of Health Sciences, Kwame Nkrumah University of Science and Technology, Kumasi, Ghana; Department of Population and Health, University of Cape Coast, Cape Coast, Ghana; Centre for Gender and Advocacy, Takoradi Technical University, Takoradi, Ghana; College of Public Health, Medical and Veterinary Sciences, James Cook University, Australia; School of Clinical Medicine, University of New South Wales Sydney, Sydney, Australia

**Keywords:** equity, Ghana, health insurance subscriptions, healthcare access, spatial distribution, women's health

## Abstract

**Background:**

This study examined the spatial distribution and factors associated with health insurance subscription among women in Ghana.

**Methods:**

We analysed a representative sample of 9380 women aged 15–49 y from the 2014 Ghana Demographic and Health Survey. Descriptive and multilevel regression analyses were performed. The study also employed spatial analysis techniques, including spatial autocorrelation, hotspot analysis, cluster and outlier analysis, as well as geographically weighted regression to explore the geographic distribution and predictors of non-subscription to health insurance.

**Results:**

The analysis revealed a moderately high prevalence of health insurance subscription among women in Ghana (62.4%). The spatial analyses indicated substantial variations in health insurance subscription across different regions in Ghana. The coastal and middle zones reported higher rates of non-subscription compared with the northern zone. We observed that young women (aged 20–24 y) had a lower likelihood of subscribing compared with adolescent girls (aged 15–19 y). Also, higher educational attainment, listening to the radio, being in a marital union and higher wealth status were positively associated with health insurance subscription.

**Conclusions:**

The study calls for targeted interventions and policies to promote equitable access to healthcare services, focusing on improving health infrastructure in coastal and middle zones, educational campaigns for individuals with lower education levels, leveraging media platforms for health insurance awareness and implementing equitable and affordable processes for individuals in poorer households.

## Introduction

Over the past three decades, universal health coverage and the protection of individuals from the burden of catastrophic out-of-pocket medical expenses have dominated discussions of global health policy.^1–3^ Given that health insurance programmes have only recently been implemented in Africa and are still changing, many questions have been raised about their efficacy in increasing the use of conventional healthcare and lowering out-of-pocket expenses for the continent's population, particularly women.^4–7^

To achieve the Sustainable Development Goal targets of universal health coverage (SDG 3.7) and reducing maternal mortality (SDG 3.1), many countries in sub-Saharan Africa have implemented health insurance policies over the last two decades.^5^ Sub-Saharan African countries such as Ghana, Kenya, Mali, Nigeria, Rwanda, South Africa, Tanzania and Zimbabwe, among others have implemented health insurance schemes that seek to improve access to healthcare for their populace.^6^ Currently, several of the programmes encompass central and local government employees and their families, while other programmes apply to the entire community.^4^ However, enrolment is significantly lower than anticipated in several countries where health insurance is seen as a pro-poor intervention.^7^ Health insurance has, however, increased access to high-quality healthcare services and offered the public financial risk protection, paying particular attention to the vulnerable and women of reproductive age.^8^

In Ghana, women face significant challenges regarding healthcare access.^9^ Access to quality health insurance has been shown to have an impact on households by improving health, especially for women who frequently require maternal and child health services, reducing the risk of health shocks and lowering out-of-pocket medical costs.^4^ Although Ghana's national health insurance programme is regarded as one of the best in sub-Saharan Africa at increasing citizens' access to healthcare services, much more must be done to encourage enrolment if the programme is to achieve the benefits that its creators had hoped for. Despite efforts to expand healthcare coverage, there are still large differences in enrolment rates across various population groups, notably among women.^10^ For instance, in 2011, 44% of men had never joined the programme compared with 31% of women.^11,5^

Notwithstanding, research by Penfold et al.^12^ indicated that additional financial and logistical barriers to the National Health Insurance Scheme (NHIS) restrict access for many women. The fact that Penfold et al.’s study is over a decade old and only focuses on the Volta and Central Regions raises doubts about its applicability to the entire country. Mensah et al’s.^13^ article gives us yet more justifications for emphasising on women. Mensah et al.^13^ revealed that increasing health insurance coverage could hasten the fulfilment of the Millennium Development Goals because insured women reported better reproductive health outcomes than uninsured women. More research is required to identify the hotspots of health insurance non-subscription to better inform policy and programmes aimed at positioning Ghana towards the attainment of universal health coverage through sustainable health financing. As a result, this study examined the spatial distribution and factors associated with health insurance subscriptions among women in Ghana. This study will encourage stakeholders to know those areas with higher proportions of women uninsured for targeted interventions.

## Ghana’s National Health Insurance Scheme

The origin of the NHIS can be traced back to a campaign pledge made by the incoming New Patriotic Party during the 2000 elections in Ghana.^14^ This pledge aimed to eliminate financial obstacles hindering access to healthcare services. Subsequently, in September 2003, the Parliament of the Republic of Ghana passed Act 650, which officially established the NHIS as a framework for healthcare regulation.^13^

Under the provisions of Act 650, the NHIS created a dedicated governing body known as the National Health Insurance Authority, which was responsible for overseeing the healthcare system. This overseeing included the accreditation of healthcare providers, negotiation of contribution rates with various schemes, management of the National Health Insurance Fund and the issuance of membership cards.^14^

This revamped healthcare system ensured that all accredited healthcare providers offer a comprehensive minimum package of services. These services encompass a wide range, including general outpatient and inpatient care at accredited facilities, oral health, eye care, handling emergencies and maternity care, encompassing prenatal care, normal delivery and complex deliveries.^11,13,14^ However, it is essential to note that certain specialised treatments, such as HIV retroviral drugs, assisted reproduction and cancer treatment, are not covered by this minimum package.^14^

The NHIS also extends its coverage to a range of diseases, including but not limited to malaria, diarrhoea, select respiratory infections, skin diseases, hypertension, asthma and diabetes, among others.^14,15^ This comprehensive coverage significantly contributes to the overall improvement of healthcare accessibility and the reduction of financial barriers, aligning with the original intention behind the NHIS. Over the years, the NHIS has gone through some revisions, notably the introduction of the free maternal healthcare policy and Act 852, which make membership of the NHIS mandatory and operational at the individual level.^14^ The National Health Insurance Authority administers the NHIS while individuals aged 18–69 y are mandated to pay a premium.^14,15^

## Materials and Methods

### Data source and study design

We analysed secondary data from the 2014 Ghana Demographic and Health Survey (GDHS). The data was extracted from the individual recode file containing responses from 9396 women of reproductive age (15–49 y). The DHS is a nationally representative survey conducted every 5 y in >90 low- and middle-income countries worldwide.^16^ The GDHS employed a cross-sectional design, with respondents sampled using a two-staged cluster sampling technique.^17,18^ The detailed sampling methodology has been published elsewhere in the literature.^17,18^ Pretested and validated questionnaires were used to collect data from the respondents. Trained data collectors were used for the survey. Our study analysed a weighted sample of 9380 women aged 15–49 y with complete observations on variables of interest. The dataset used can be accessed at https://dhsprogram.com/data/dataset/Ghana_Standard-DHS_2014.cfm?flag=1. We drafted this paper as per the Strengthening Reporting of Observational Studies in Epidemiology (STROBE) guidelines.^19^

### Variables

Health insurance subscription was the dependent variable in our study. This variable was measured in the DHS using the question: ‘Are you covered by any health insurance?’ Examples of the health insurance policies the respondents subscribed to include (but are not limited to) mutual health insurance, national or district health insurance, employer-based health insurance, social security and privately purchased commercial insurance. In the DHS, the response to the question was ‘0=no’ and ‘1=yes’. We utilised this definite response in our final analysis. Previous studies using the DHS employed the same categorisation.^5,20–22^

Based on the review of pertinent literature,^5,20–22^ we included 13 explanatory variables in our study. Also, these variables were available in the 2014 GDHS dataset. The variables include women's age, level of education, marital status, religion, working status, parity, exposed to watching television, exposed to listening to the radio, exposed to reading newspapers or magazines, wealth index, sex of household head, place of residence and region. We grouped the variables into individual level and contextual level with reference to literature that used the DHS dataset to examine health insurance subscription.^5,20–22^ The categories of the variables can be found in Table 1.

**Table 1.** Prevalence and distribution of health insurance subscription across explanatory variables (n=9380)

	Weighted		Health insurance subscription	
Variable	Sample	Percentage (%)	Yes (%) 62.4 [CI=60.3, 64.4]	p
Women's age (y)				<0.001
15–19	1620	17.3	59.4 [56.2, 62.6]	
20–24	1609	17.2	58.0 [54.5, 61.5]	
25–29	1602	17.1	65.9 [62.8, 68.9]	
30–34	1371	14.6	66.2 [62.8, 69.4]	
35–39	1294	13.8	66.2 [62.0, 70.1]	
40–44	1027	10.9	59.8 [55.4, 64.1]	
45–49	857	9.1	60.8 [55.3, 66.0]	
Level of education				<0.001
No education	1790	19.1	61.5 [57.8, 65.1]	
Primary	1670	17.8	56.3 [53.0, 59.6]	
Secondary	5325	56.8	63.1 [60.7, 65.5]	
Higher	596	6.3	74.9 [69.8, 79.4]	
Marital status				<0.001
Never in union	3087	32.9	58.0 [55.5, 60.5]	
Married	3962	42.2	70.0 [67.5, 72.3]	
Cohabiting	1351	14.4	57.2 [53.6, 60.8]	
Widowed	253	2.7	59.9 [50.4, 68.7]	
Divorced	281	3.0	47.9 [39.1, 56.9]	
Separated	446	4.8	51.3 [45.4, 57.3]	
Religion				<0.001
Christianity	7519	80.2	61.4 [59.2, 63.6]	
Islamic	1420	15.1	70.9 [67.2, 74.3]	
Traditionalist	188	2.0	57.5 [49.9, 64.8]	
Others or none	253	2.7	47.6 [40.8, 54.5]	
Current working status				0.728
No	2490	26.5	62.7 [60.1, 65.2]	
Yes	6890	73.5	62.3 [60.0, 64.5]	
Parity				<0.001
Zero	2931	31.3	59.5 [56.6, 62.3]	
One birth	1326	14.1	66.5 [63.4, 69.4]	
Two births	1315	14.0	67.4 [63.4, 71.1]	
Three births	1125	12.0	64.3 [59.9, 68.4]	
Four or more births	2683	28.6	60.3 [57.2, 63.3]	
Exposed to watching television				0.105
No	2202	23.5	60.0 [56.3, 63.7]	
Yes	7178	76.5	63.1 [61.0, 65.2]	
Exposed to listening to radio				0.006
No	1468	15.7	57.7 [53.7, 61.5]	
Yes	7912	84.3	63.3 [61.1, 65.4]	
Exposed to reading newspaper or magazine				0.004
No	7611	81.1	61.3 [59.1, 63.5]	
Yes	1769	18.9	67.0 [63.4, 70.4]	
Wealth index				<0.001
Poorest	1510	16.1	64.6 [60.2, 68.7]	
Poorer	1636	17.4	57.6 [53.1, 62.1]	
Middle	1934	20.6	58.9 [55.2, 62.5]	
Richer	2115	22.6	61.8 [58.8, 64.7]	
Richest	2185	23.3	68.1 [64.1, 71.8]	
Sex of household head				<0.001
Male	5752	61.3	64.6 [62.3, 66.9]	
Female	3628	38.7	58.8 [56.1, 61.5]	
Place of residence				0.218
Urban	5039	53.7	63.6 [61.0, 66.1]	
Rural	4341	46.3	61.0 [57.7, 64.2]	
Region				<0.001
Western	1037	11.1	64.9 [59.8, 69.7]	
Central	934	10.0	48.2 [43.0, 53.4]	
Greater Accra	1891	20.2	58.9 [53.9, 63.7]	
Volta	720	7.7	70.1 [64.3, 75.4]	
Eastern	874	9.3	67.9 [61.9, 73.3]	
Ashanti	1795	19.1	52.6 [47.5, 57.7]	
Brong Ahafo	768	8.2	76.3 [71.1, 80.8]	
Northern	786	8.4	70.7 [63.1, 77.3]	
Upper East	358	3.8	68.9 [63.8, 73.6]	
Upper West	213	2.3	85.3 [76.3, 91.2]	

### Statistical analyses

#### Descriptive and multilevel regression analysis

We used Stata software version 17.0 (Stata Corporation, College Station, TX, USA) to perform the descriptive and inferential analyses. We used percentages to present the proportion of health insurance subscription among the women. Later, we examined the distribution of health insurance subscription across the explanatory variables using cross-tabulation. Pearson’s χ^2^ test of independence was used to examine the explanatory variables significantly associated with health insurance subscription at p<0.05. We used multilevel binary logistic analysis to examine the factors associated with health insurance subscription. We used four models to examine the factors. Model O was an empty model with no explanatory variables. Model I, Model II and Model III included the individual level, contextual level and all explanatory variables, respectively. The regression results were presented in two formats showing the fixed effect and random effects. The fixed effect results showed the association between health insurance subscription and explanatory variables. We presented the fixed effect results using adjusted odds ratio (AOR) with their respective 95% confidence intervals (CIs). Statistical significance was set at p<0.05. The random effects denoted the measure of variation in health insurance subscription based on 427 primary sampling units or clusters (measured by intra-cluster correlation). We included region in the regression to control for the effect of region in the model. The cluster variable was included in the multilevel models to take into consideration the clustering of the dataset. The random effect model results aided in assessing the fitness of the models as well as comparing the various models to ascertain the best-fitted model for the study. The model with the least Akaike Information Criterion was selected as the best fitted model and its results were interpreted and discussed. We also weighted all the analyses to adjust for disproportionate sampling and non-response.^16^

#### Spatial analysis

The dependent variable was health insurance subscription but for the purpose of spatial analysis, the dependent variable was recoded into no health insurance subscription (1) and health insurance subscription (0) to. The district names were attached to the coordinates of the surveyed data using the join tool in ArcMap version 10.5. This approach aimed to attach the district names to the coordinates of the surveyed data. The coordinates had cluster numbers serving as a proxy for linking the district names to the actual surveyed data. The purpose of this joining was to enable agglomerate responses at the district level, which would be used for spatial analysis. Specifically, adding the district names to the surveyed data aided in generating proportions of those with no health insurance subscription in Ghana. The estimated proportions of no health insurance subscription were joined to the district shapefiles for the spatial analysis.

The initial spatial analytical tool used was spatial autocorrelation, which explores the geographic distribution of no health insurance subscription in Ghana at the district level. The spatial autocorrelation's output tells whether the data are dispersed, random or clustered. Because spatial analysis is used to study the distribution of the phenomenon being studied, the study went a step further to visualise the geographic distribution of no health insurance subscription in Ghana using the Getis-Ord G hotspot analytical tool. The hotspot analysis was employed to identify statistically significant variations in the distribution of no health insurance subscriptions in Ghana at the district level. Lastly, the cluster and outlier analysis were used to detect outlier districts to inform appropriate policies and programmes. The last and not least employed spatial analytical tool in this study was the geographically weighted regression (GWR).

This analysis required providing explanatory variables that can explain the observed distribution of no health insurance subscription. Therefore, explanatory variables that could affect people for not subscribing to health insurance were extracted. Some of the explanatory variables were the proportion of respondents who reside rural areas, read newspapers, listened to the radio, watched television, belonged to the poorest wealth quintile, unemployed, had no formal education, were Christians, and had four or more children. Prior to this analysis, an ordinary least squares (OLS) test was conducted to identify significant predictors of no health insurance subscription. Three explanatory variables were found to be significant predictors of no health insurance subscription, and these were used to run the GWR analysis. The GWR model generates local regression coefficients for each observation by considering the neighbouring observations. The formula for GWR is as follows:


\begin{equation*}
Yi = \beta 0\left( {ui,vi} \right) + \ \mathop \sum \limits_{k = 1}^p \beta k\left( {ui,vi} \right)Xki + \ \varepsilon i
\end{equation*}

where:

- Y*i* is the observed value of the dependent variable for the *i*th location;
-
$\beta 0( {ui,vi} )$ is the local intercept coefficient for the *i*th location, which considers the spatial variation;
-
$\beta k( {ui,vi} )$ is the local coefficient of the *k*th independent variable for the *i*th location;
-
$ Xki $ is the observed value of the *k*th independent variable for the *i*th location; and
-
$ \varepsilon i $ represents the error term for the *i*th location.

In this formula, the $ \beta 0( {ui,vi} ) $ and $\beta k( {ui,vi} )$ coefficients are modelled as spatially varying parameters. They change for each location due to the inclusion of the coordinates u_i_ and v_i_ as inputs. These coordinates reflect the geographic position of the observation. The results from all the analyses are presented in tables, charts and maps.

## Results

### Distribution of health insurance subscriptions across the explanatory variables

Table 1 presents the proportions of health insurance subscriptions across various explanatory variables. The overall proportion of health insurance subscription was moderately high at 62.4%. Notably, women in the Upper West Region reported the highest proportion at 85.3%. Within specific age groups, women aged 30–34 and 35–39 y had the highest proportions. The analysis revealed significantly higher proportion of health insurance coverage among women with a higher level of education (74.9%), those who were married (70%) and those who identified as Islamic (70.9%). The proportion of health insurance coverage was highest among women with two birth (two children) (67.4%). The wealth index displayed a clear association, with the wealthiest individuals having the highest subscription proportion of 68.1%, while the poorest individuals had a prevalence of 64.6%. Additionally, the sex of the household head showed significance, with males exhibiting a higher subscription prevalence of 64.6% compared with females at 58.8%.

### Spatial distribution of no health insurance subscription

**Figure 1.**
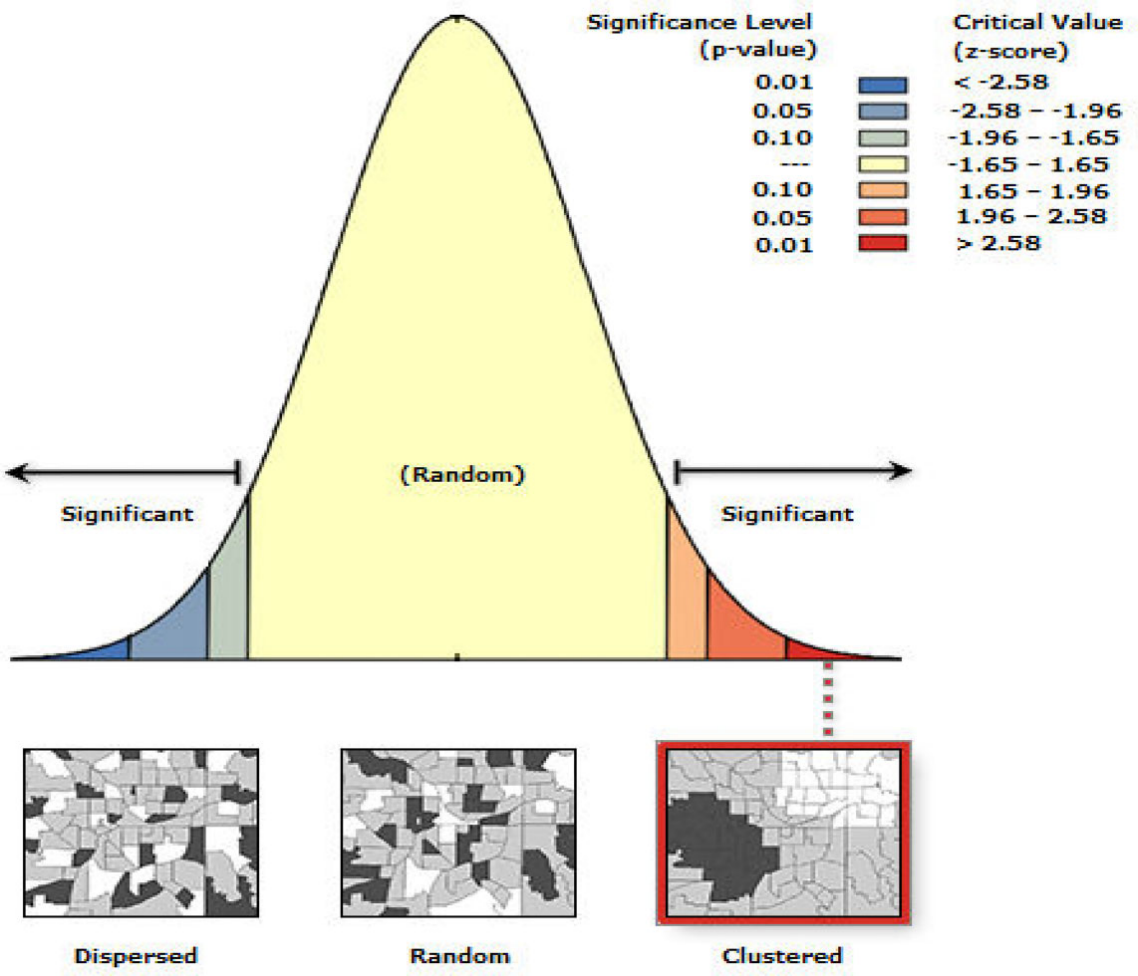
Spatial pattern of no health insurance subscription in Ghana.

Figure 1shows that the geographic distribution of no health insurance subscription was clustered. This implies that the distribution of no health insurance subscription was not random or dispersed. The result simulates further spatial analysis because the spatial autocorrelation analysis does not specify in which areas the phenomenon is clustered. Therefore, hotspot analysis was conducted to apply spatial statistics in identifying areas where there was clustering of no health insurance subscription in Ghana at the district level.

**Figure 2.**
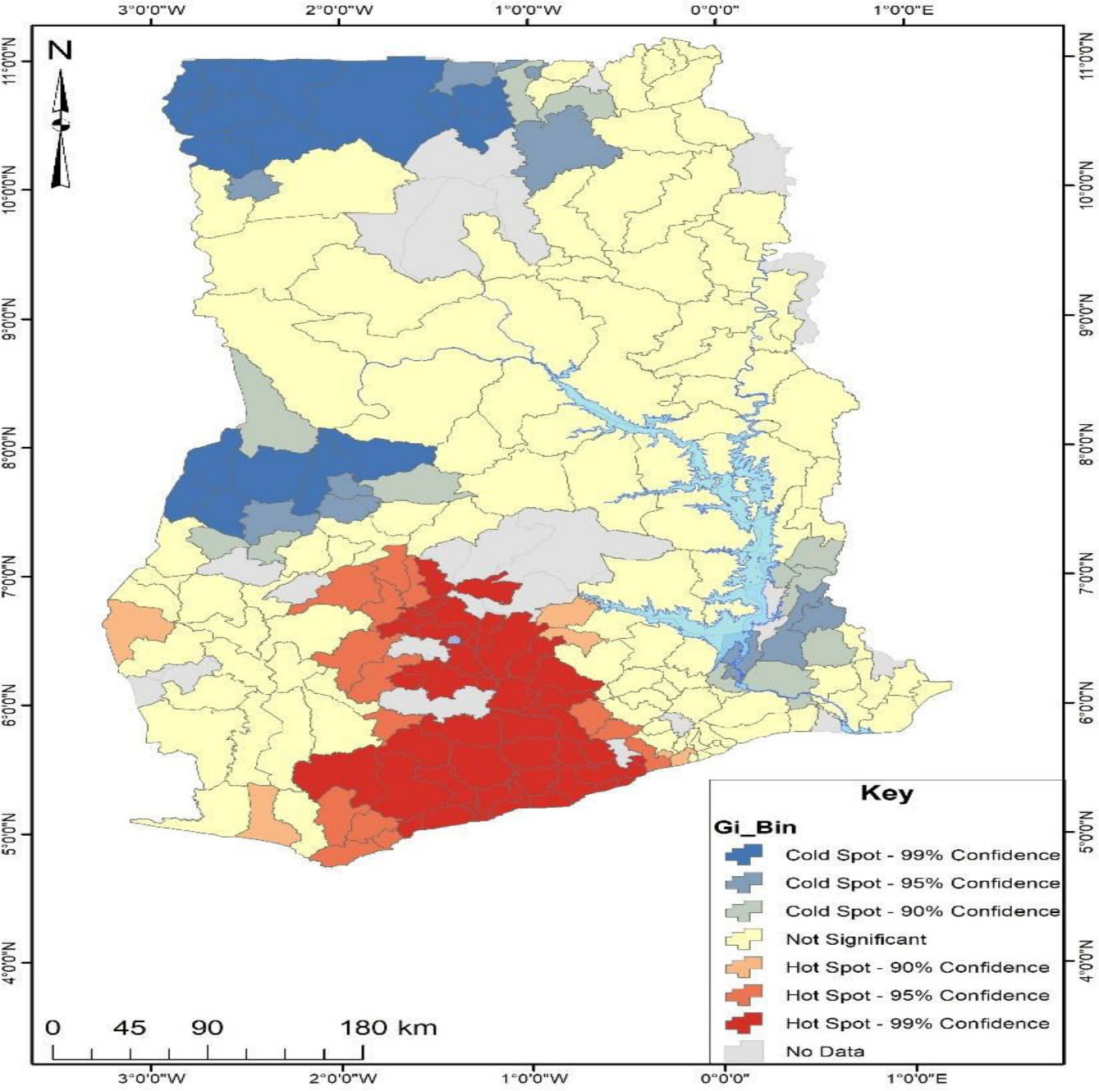
Hotspot analysis of no health insurance subscription in Ghana.

Figure 2 presents the results of the Getis-Ord Gi Hotspot analysis, indicating three distinct colours (shades of red, blue and yellow). The red and blue areas display hotspot and cold spot areas, respectively, but at different confidence levels ranging from 90% to 99%. The deeper the colour, the higher the confidence level of the prevalence of the phenomenon being studied. The area displayed in yellow had no statistical significance. From Figure 2, districts in the south-central of Ghana had a high prevalence of no health insurance subscription. Some of these districts were Cape Coast Metropolis, Sekyere South, Assin South, Bosomtwe and Atwima Nwabiagya. On the other hand, some districts were found to have a significantly low prevalence of no health insurance subscription and were in the northwest and west of the middle belt in Ghana. Some districts with a low incidence of no health insurance subscription were Sissala East, Nadowli, Wa Municipal, Tain and Kintampo South.

**Figure 3.**
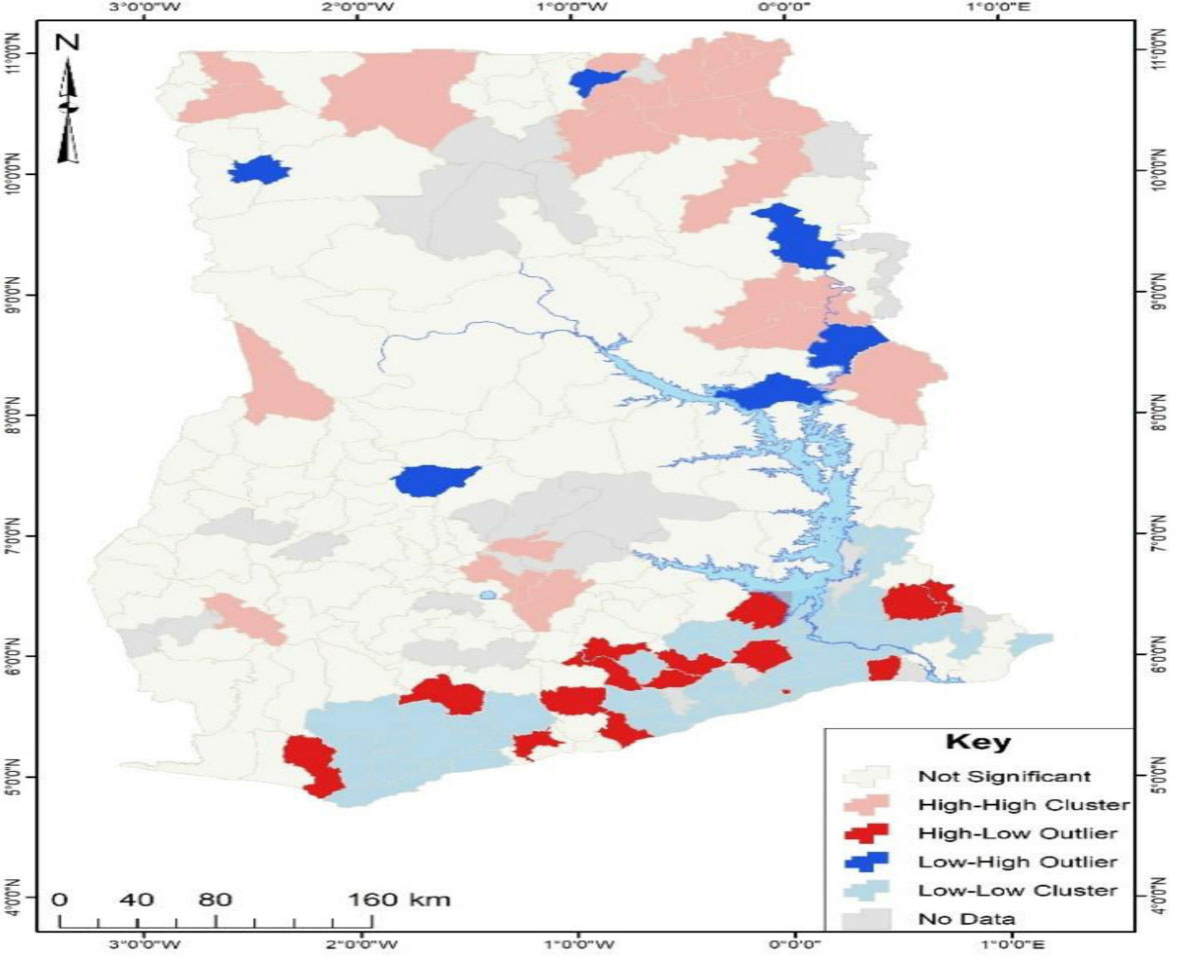
Cluster and outlier analysis of no health insurance subscription in Ghana.

The Get-Ord Gi Hotspot analysis fails to factor in outliers in the dataset. Therefore, the Anselin Cluster and Outlier analytical tool was used to identify outliers in the no health insurance subscription in Ghana at the district level. The cluster and outlier results (Figure 3) revealed that districts displayed in red, such as Ada West, Nzema East, Upper Manya Krobo and Adaklu, had a high prevalence of no health insurance subscription but were surrounded by districts with a relatively low prevalence of no health insurance subscription. Conversely, the results presented in Figure 3 show that the districts in blue, such as Bolgatanga Municipal, Yendi Municipal, Krachi Nchumuru and Nkoranza South, had a low incidence of no health insurance subscription but were surrounded by districts with a relatively high prevalence of no health insurance subscription.

### Spatial predictors of no health insurance subscription in Ghana

**Figure 4.**
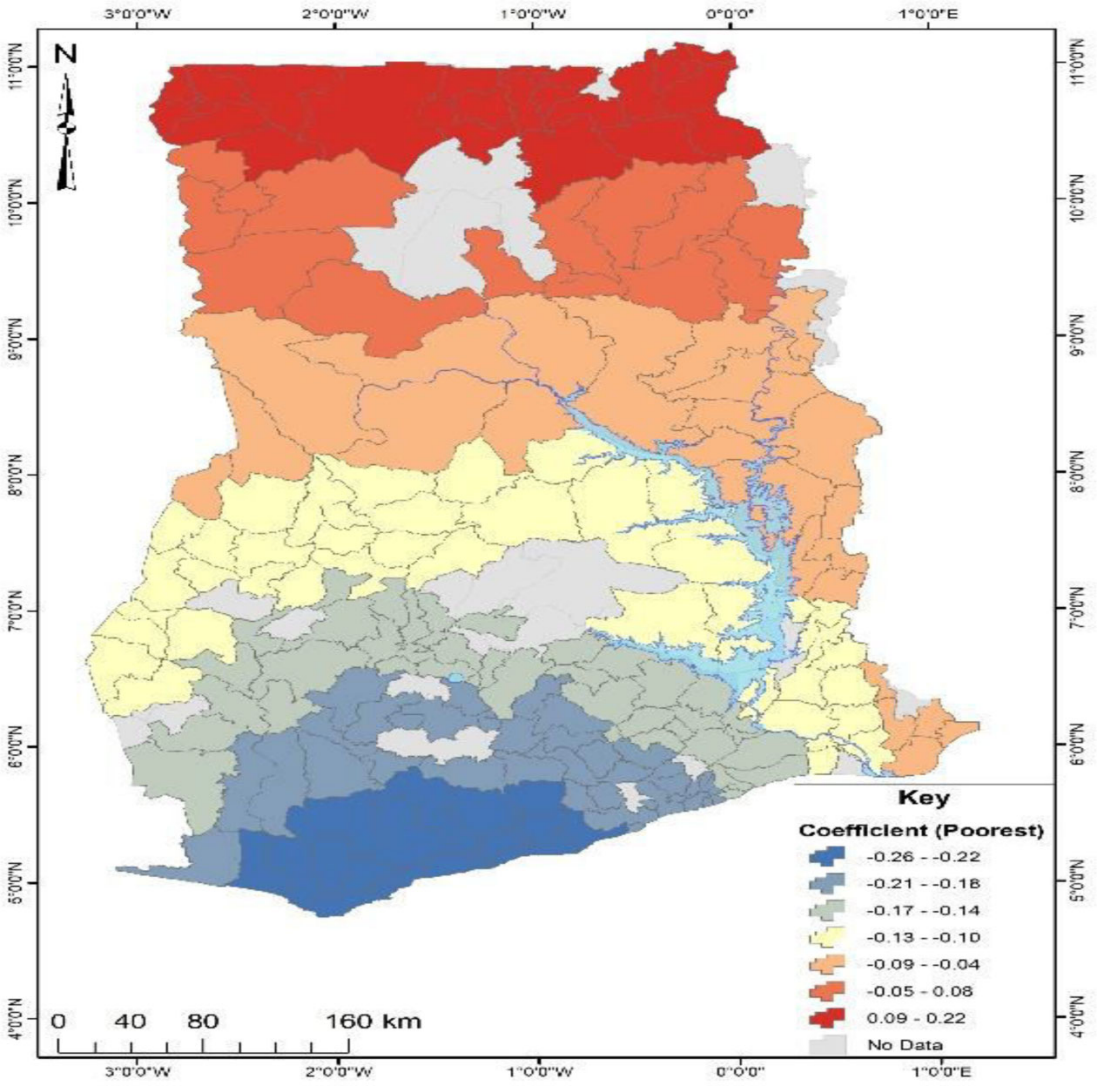
Predictive power of poorest wealth status for no health insurance subscription in Ghana.

**Figure 5.**
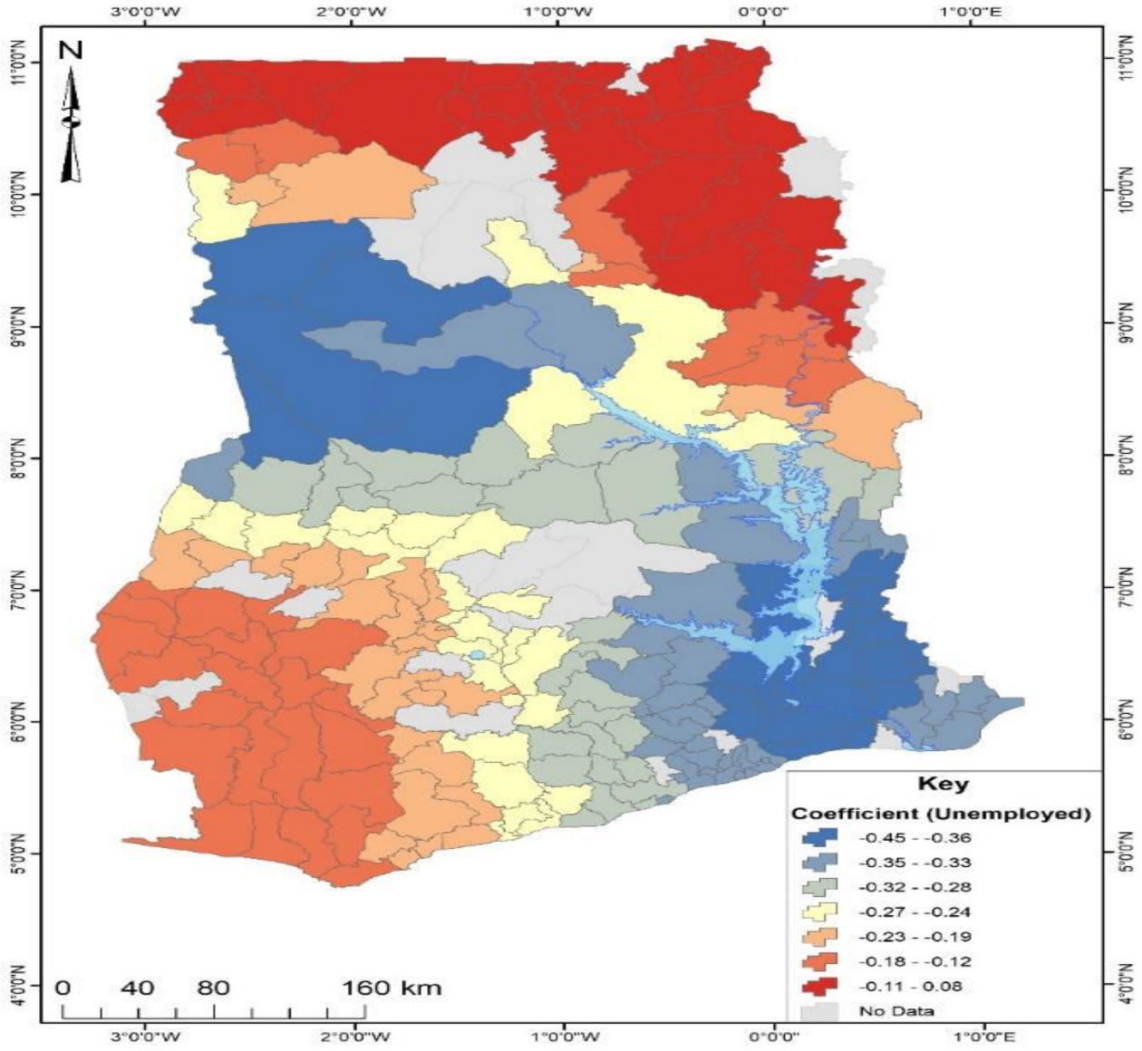
Predictive power of unemployment status for no health insurance subscription in Ghana.

**Figure 6.**
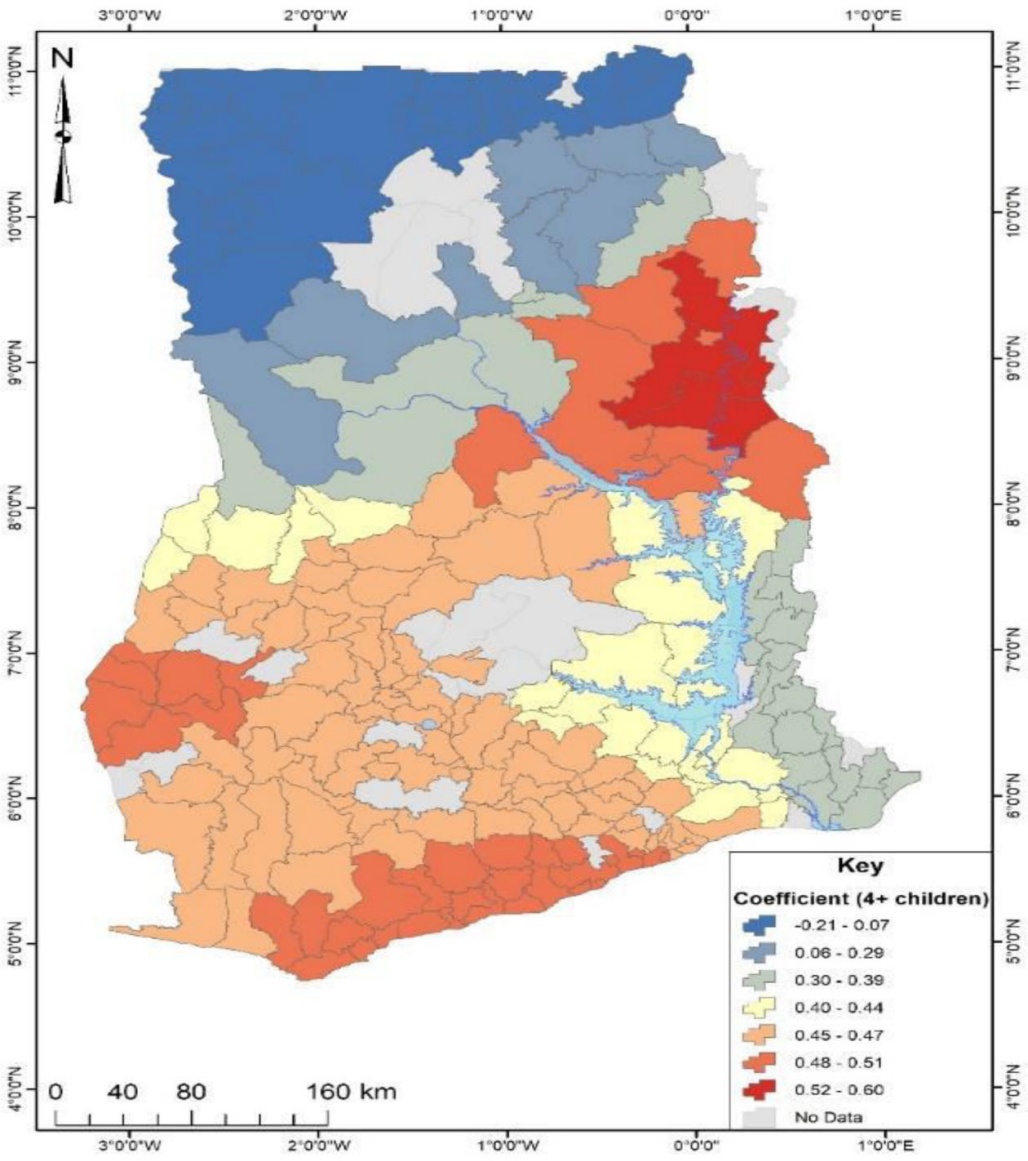
Predictive power of four or more children for no health insurance subscription in Ghana.

An in-depth investigation was conducted to determine the factors responsible for Ghana's observed geographical distribution of no health insurance subscription. The study employed GWR analysis, which required the identification of significant factors explaining the distribution of no health insurance subscription in Ghana using OLS analysis. As presented in Table 2, the OLS results revealed that out of nine factors, only three (being unemployed, being in the poorest wealth index and multiparity) could explain the spatial distribution of no health insurance subscription across Ghana. The three significant predictors from the OLS results were used to run the GWR analysis, and the model parameters are presented in Table 4. The GWR model, which estimates the response of the dependent variable to independent variables based on locational effects, had an R^2^ value of 32% (Table 3). Figures 4–6 depict the spatial predictive power of the explanatory variables on no health insurance subscription in Ghana, with red areas having a strong predictive power, while blue areas have a low predictive power of the explanatory variable.

**Table 2.** OLS results of factors that determine no health insurance subscription in Ghana

Variable	Coefficient	Standard Error	t-Statistic	Probability	Robust_SE	Robust_t	Robust_Pr [b]
Intercept	29.085	14.775	1.968	0.051	13.636	2.133	0.034*
Rural	0.009	0.044	0.195	0.845	0.039	0.216	0.829
Newspaper (not at all)	−0.021	0.155	−0.135	0.893	0.149	−0.140	0.889
Radio (not at all)	−0.031	0.112	−0.278	0.782	0.107	−0.289	0.773
Tv (not at all)	0.144	0.089	1.619	0.107	0.097	1.485	0.139
Poorest	−0.246	0.080	−3.078	0.002*	0.085	−2.883	0.004*
Unemployed	−0.279	0.120	−2.324	0.021*	0.118	−2.368	0.018*
No formal education	0.016	0.122	0.133	0.895	0.116	0.140	0.889
Christianity	0.052	0.072	0.727	0.468	0.073	0.720	0.472
4+ children	0.348	0.132	2.639	0.009*	0.145	2.398	0.017*

Source: GDHS, 2014.

*Significant variable.

**Table 3.** OLS diagnostics

Number of observations	190	Akaike's Information Criterion (AICc)	−96.171
Multiple R-Squared	0.352	Adjusted R-Squared	0.335
Joint F-Statistic	20.022	Prob(>F), (5184) degrees of freedom	0.000000*
Joint Wald Statistic	107.666	Prob(>χ^2^), (5) degrees of freedom	0.000*
Koenker (BP) Statistic	23.211	Prob(>χ^2^), (5) degrees of freedom	0.000*
Jarque-Bera Statistic	4.349	Prob(>χ^2^), (2) degrees of freedom	0.114

Source: GDHS, 2014.

*Significant variable.

**Table 4.** GWR model parameters for no health insurance subscription in Ghana

Explanatory variables	Rural, newspaper (not at all), radio (not at all), TV (not at all), poorest, unemployed, no formal education, Christianity, 4+ children
Bandwidth	178 601.283
Residual squares	45 877.156
Effective number	20.82
Sigma	16.323
AICc	1638.962
R^2^	0.316
R^2^ adjusted	0.238

Source: GDHS, 2014.

From Figure 4, the coefficient of the poorest wealth status ranged from −26% to 22%. This suggested that the poorest wealth status had positive and negative predictive effects in relation to no health insurance subscription. Areas in red suggest that a unit increase in those respondents with the poorest wealth status accounted for a 22% increase in no health insurance subscription. This pattern was observed in the northern parts of Ghana. On the other hand, a unit increase in those respondents with the poorest wealth status accounted for a 26% decrease in no health insurance subscription. This pattern was observed in the central and southern parts of Ghana.

From Figure 5, the coefficient of unemployed respondents ranged from −45% to 8%. This indicated that unemployment status had positive and negative predictive effects on no health insurance subscription. Areas in red suggest that a unit increase in respondents’ unemployment status accounted for an 8% increase in no health insurance subscription. This pattern was observed in the northern parts of Ghana. However, a unit increase in respondents’ unemployment status accounted for a 45% decrease in no health insurance subscription. This pattern was observed in the southeast and northwest of Ghana.

From Figure 6, the coefficient of respondents with four or more children ranged from −21% to 60%. This indicated that respondents with four or more children had positive and negative predictive effects on no health insurance subscription. Areas in red indicated that a unit increase in respondents with four or more children accounted for a 60% increase in no health insurance subscription. This pattern was observed in the north-eastern parts of Ghana. Nevertheless, a unit increase in respondents with four or more children accounted for a 21% decrease in no health insurance subscription. This pattern was observed in the northern parts of Ghana.

### Factors associated with health insurance subscription among women in Ghana

Table 5 shows the results of the multilevel regression analyses used to examine the factors associated with health insurance subscription among women in Ghana. Compared with adolescent girls (aged 15–19 y), young women (aged 20–24 y) had a significantly lower likelihood of subscribing to health insurance (AOR: 0.80, 95% CI 0.64 to 0.99). Women with secondary education (AOR: 1.50, 95% CI 1.25 to 1.80) and higher education (AOR: 2.34, 95% CI 1.65 to 3.33) exhibited significantly higher odds of health insurance subscription compared with those with no education.

**Table 5.** Factors associated with health insurance subscription among women in Ghana

Variable	Model O	Model I (AOR) AOR [95% CI]	Model II (AOR) AOR [95% CI]	Model III (AOR) AOR [95% CI]
Fixed effect results				
Women's age (y)				
15–19		1.00		1.00
20–24		0.79* [0.64, 0.99]		0.80* [0.64, 0.99]
25–29		0.96 [0.76, 1.22]		0.95 [0.74, 1.21]
30–34		1.02 [0.77, 1.35]		0.99 [0.74, 1.31]
35–39		1.09 [0.79, 1.49]		1.05 [0.77, 1.44]
40–44		0.87 [0.62, 1.21]		0.86 [0.62, 1.20]
45–49		0.94 [0.67, 1.32]		0.95 [0.67, 1.34]
Level of education				
No education		1.00		1.00
Primary		1.03 [0.87, 1.23]		1.07 [0.89, 1.28]
Secondary		1.51*** [1.27, 1.79]		1.50*** [1.25, 1.80]
Higher		2.59*** [1.86, 3.59]		2.34*** [1.65, 3.33]
Marital status				
Never in union		1.00		1.00
Married		2.54*** [1.96, 3.27]		2.30*** [1.74, 3.04]
Cohabiting		1.36* [1.06, 1.74]		1.31* [1.02, 1.70]
Widowed		1.81* [1.14, 2.87]		1.69* [1.07, 2.66]
Divorced		0.96 [0.63, 1.48]		0.91 [0.58, 1.43]
Separated		1.07 [0.75, 1.51]		1.08 [0.76, 1.53]
Religion				
Christianity		1.00		1.00
Islamic		1.49*** [1.22, 1.82]		1.40*** [1.15, 1.71]
Traditionalist		0.98 [0.70, 1.36]		0.92 [0.66, 1.30]
Others or none		0.69* [0.48, 0.99]		0.70 [0.49, 1.00]
Parity				
Zero		1.00		1.00
One birth		1.17 [0.91, 1.50]		1.19 [0.93, 1.53]
Two births		1.07 [0.78, 1.48]		1.12 [0.81, 1.55]
Three births		0.82 [0.63, 1.09]		0.88 [0.66, 1.17]
Four or more births		0.69** [0.53, 0.91]		0.77 [0.58, 1.03]
Exposed to reading newspaper or magazine				
No		1.00		1.00
Yes		1.10 [0.91, 1.34]		1.05 [0.87, 1.27]
Exposed to listening to radio				
No		1.00		1.00
Yes		1.27** [1.06, 1.52]		1.22* [1.02, 1.46]
Sex of household head				
Male			1.00	1.00
Female			0.81*** [0.73, 0.91]	1.03 [0.89, 1.19]
Wealth index				
Poorest			1.00	1.00
Poorer			1.19 [0.94, 1.50]	1.12 [0.88, 1.42]
Middle			1.60*** [1.27, 2.02]	1.38* [1.08, 1.77]
Richer			2.60*** [1.94, 3.50]	1.95*** [1.41, 2.69]
Richest			4.23*** [3.12, 5.72]	2.60*** [1.87, 3.62]
Region				
Western			1.00	1.00
Central			0.55*** [0.40, 0.75]	0.52*** [0.38, 0.72]
Greater Accra			0.50*** [0.36, 0.70]	0.51*** [0.36, 0.72]
Volta			1.74** [1.20, 2.52]	1.71** [1.17, 2.49]
Eastern			1.39 [0.97, 1.99]	1.38 [0.95, 2.01]
Ashanti			0.50*** [0.35, 0.72]	0.49*** [0.34, 0.72]
Brong Ahafo			2.42*** [1.71, 3.45]	2.26*** [1.58, 3.25]
Northern			2.21*** [1.48, 3.28]	1.99*** [1.33, 2.99]
Upper East			2.15*** [1.48, 3.15]	1.85** [1.25, 2.75]
Upper West			6.51*** [3.37, 12.58]	5.48*** [2.82, 10.67]
Random effect model				
PSU variance (95% CI)	0.678 [0.556, 0.827]	0.705 [0.575, 0.863]	0.465 [0.352, 0.614]	0.474 [0.356, 0.629]
ICC	0.171	0.176	0.124	0.126
Wald χ^2^	Reference	341.34 (<0.001)	260.92 (<0.001)	519.70 (<0.001)
Model fitness				
Log-likelihood	−5883.2927	−5677.6257	−5717.2925	−5577.7356
AIC	11 770.59	11 405.25	11 466.58	11 233.47
BIC	11 784.88	11 583.91	11 580.93	11 512.18
N	9380	9380	9380	9380
Number of clusters	427	427	427	427

Abbreviations: AIC, Akaike's information criterion; AOR, adjusted odds ratio; ICC, intra-class correlation; PSU, primary sampling unit; BIC, Bayesian Information Criterion.

*p< 0.05; **p<0.01; ***p<0.001; 1.00=reference category.

The odds of health insurance subscription was significantly higher among married women (AOR: 2.30, 95% CI 1.74 to 3.04), cohabiting women (AOR: 1.31, 95% CI 1.02 to 1.70) and widowed women (AOR: 1.69, 95% CI 1.07 to 2.66) compared with women who have never been in a marital union. Also, those who identified as Islamic (AOR: 1.40, 95% CI 1.15 to 1.71) showed significantly higher odds of health insurance subscription compared with Christians. The likelihood of health insurance subscription was high among those exposed to listening to the radio (AOR: 1.22, 95% CI 1.02 to 1.46) compared with those not exposed, and among women in the richest wealth index (AOR: 2.60, 95% CI 1.87 to 3.62) than among those in the poorest wealth index. There were also some regional variations with women in the Ashanti region reporting the least odds of having health insurance subscription (AOR: 0.49, 95% CI 0.34 to 0.72), while those in the Upper West region (AOR: 5.48, 95% CI 2.82 to 10.67) reported the highest odds.

## Discussion

The present study examined the spatial distribution and factors associated with health insurance subscription among women in Ghana. Women in Ghana had moderately high health insurance subscription (62.4%). The observed proportion of health insurance subscription is higher than the prevalence of 40.2% reported by Kumi-Kyereme and Amo-Adjei^23^ in Ghana. This difference in study findings can be explained by the recent nature of the data used for the analysis. While their study relied on the 2008 GDHS, the present study used the 2014 GDHS. It also suggests the effectiveness of the existing strategies (e.g. the compulsory enrolment of all pregnant women in the health insurance scheme implemented in Ghana to improve health insurance subscription). Nevertheless, the findings affirm Amu's^24^ assertion that health insurance has failed to achieve 100% subscription. The perceived high cost of health insurance premiums might explain the inability of Ghana to achieve 100% subscription.^4^ A qualitative study conducted in Ghana^25^ has shown that the perceived poor quality of drugs and healthcare services explain the country's inability to achieve 100% health insurance subscription.

Our spatial analyses revealed that there were substantial differences in terms of health insurance subscription. From the results, it is indicative that individuals in the coastal (i.e. Cape Coast Metropolis, Assin South) and middle zone (Sekyere South, Bosomtwe and Atwima Nwabiagya) reported high non-subscription to any health insurance scheme compared with those in the northern zone (i.e. Sissala East, Nadowli, Wa Municipal, Tain and Kintampo South). The hotspot analysis also showed similar patterns. This is consistent with Kumi-Kyereme and Amo-Adjei's^23^ study, which found a higher subscription among those in the northern zone compared with those in the coastal or middle zones. The results suggest that poverty, unemployment and high parity justify the non-subscription in the identified hotspots. Nevertheless, we postulate that this variation could reflect the concentration of health interventions and non-governmental organisations (NGOs) in the northern zone relative to the coastal and middle zones. For instance, Kwao and Amoak^26^ posit that the high concentration of NGOs in northern Ghana supports their economic livelihoods, empowering them and granting them the resources to manage their lives. It is possible that these NGOs also assist women in the northern zone to subscribe to the health insurance scheme, thereby reducing the prevalence of non-subscription compared with those in the coastal and middle zones.

Regarding factors associated with health insurance subscription, the study showed that young women (aged 20–24 y) had a significantly lower likelihood of subscribing to health insurance compared with adolescent girls (15–19 y). Our result contradicts a previous study conducted in Kenya^27^ that found the all-age groups to be more likely to subscribe to health insurance compared with those aged 15–19 y. In Ghana, adolescents are often under the protection of their guardians, who provide them with their basic needs and accessibility to healthcare. Therefore, their guardians will probably undertake their health insurance subscription on their behalf. However, young women are considered adults and are often responsible for taking their own healthcare decisions, including health insurance subscription. Another reason why people aged 15–19 y may have higher odds of subscribing to health insurance is the increased requirement by secondary and tertiary institutions to enrol their students on NHIS.

Consistent with previous literature,^4,27^ our study confirms that having higher educational attainment increases the likelihood of health insurance subscription. Higher education levels are often linked to an increased awareness of health insurance benefits, improved understanding of healthcare systems and an enhanced ability to navigate insurance processes.^28^ Relatedly, the study indicates that listening to the radio increases the likelihood of having a health insurance subscription, a result that mirrors that of previous studies.^19,29^ Women are more likely to subscribe to health insurance after being exposed to information highlighting the benefits and significance of health insurance coverage.^21^ This study's findings underscore the vital role played by mass media in disseminating and adopting health-related knowledge and policies. It, thus, emphasises the potential of media platforms in promoting awareness, understanding and active participation in healthcare initiatives.

The study reveals a significant association between marital status and health insurance subscription, with higher odds being reported among those who are currently married. These findings align with a study conducted by Ayanore et al.,^30^ which similarly demonstrated that married women are more likely to subscribe to health insurance than those who have never been in a marital union. This result suggests that being in a formal or informal union may offer social and economic support, ultimately enhancing access to health insurance. The presence of shared household resources and joint decision-making processes among married and cohabiting individuals may contribute to their increased likelihood of obtaining health insurance coverage.

Women in the richest wealth index demonstrated significantly higher odds of health insurance subscription than those in the poorest. This result corresponds with the established association between socioeconomic status and health insurance coverage.^21,23^ The observed association is attributable to the point that women with greater wealth often have better access to financial resources, enabling them to afford health insurance premiums.^21^ There is, therefore, a need for Ghana to review its health insurance subscription processes and premiums to be tailored to meet the financial situation of those in poorer wealth index households.

## Implications for policy and practice

The observed spatial variation in non-subscription to health insurance suggests that targeted interventions and policy strategies are needed to promote equitable access to healthcare services across different zones in the country. The government must

improve health infrastructure and service provision in the coastal and middle zones, where higher rates of non-subscription were identified. The positive association between higher educational attainment and health insurance subscription emphasises the importance of educational campaigns and initiatives targeting individuals with lower education levels. Policymakers should focus on promoting health insurance awareness and understanding among these populations, highlighting the benefits and importance of coverage. Media platforms, especially radio, should continue to be leveraged to disseminate information about health insurance, as they have effectively influenced subscription decisions. Also, the association between wealth index and health insurance subscription highlights the socioeconomic disparities in coverage. Therefore, efforts should be made to ensure that health insurance processes and premiums are equitable and affordable for individuals in poorer wealth index households. This may involve targeted subsidies or financial assistance programmes to facilitate enrolment and reduce financial barriers for these populations.

## Strengths and limitations

Using a large nationally representative dataset like the GDHS grants us the statistical power to extrapolate the findings to the entire country. Also, by incorporating spatial analysis in this study, we were able to identify hotspots and clusters of policy interest. Hence, policymakers can better target resources, interventions and services to areas with the greatest needs or disparities. However, there were some limitations. The cross-sectional nature of the GDHS does not allow us to establish a cause-and-effect relationship among the factors identified. Key factors (e.g. perceptions about health insurance, quality of health insurance, cultural beliefs) that have the potential to influence health insurance subscription could not be assessed because of their absence in the secondary data used.

## Conclusion

Our study illuminates the relevance of spatial distribution in targeting key subpopulations that may require special interventions to enhance their likelihood of subscribing to health insurance. The study further concludes that actions and initiatives aimed at improving health insurance subscription among women in Ghana must target young women, those with low educational attainment and address wealth-related inequalities. Mass media can be leveraged as a tool to educate the population about the need for and benefits that come from subscribing to health insurance.

## Data Availability

The dataset is freely accessible at https://dhsprogram.com/data/dataset/Ghana_Standard-DHS_2014.cfm?flag=1.
